# Reinforcement of Thermo-Compressed Sodium Alginate Films with Calcium Alginate Powder

**DOI:** 10.3390/md24040142

**Published:** 2026-04-17

**Authors:** Prasong Srihanam, Wilaiwan Simchuer, Vanseng Chounlamany, Kesiny Phomkeona, Phengxay Deevanhxay, Yodthong Baimark

**Affiliations:** 1 Biodegradable Polymers Research Unit, Department of Chemistry and Centre of Excellence for Innovation in Chemistry (PERCH-CIC), Faculty of Science, Mahasarakham University, Maha Sarakham 44150, Thailand; 2 Faculty of Science and Technology, Loei Rajabhat University, Mueang District, Loei 4200, Thailand; wilaiwan.sim@lru.ac.th; 3 Department of Chemistry, Faculty of Natural Sciences, National University of Laos, Vientiane 7322, Laos; v.chounlamany@nuol.edu.la (V.C.); k.phomkeona@nuol.edu.la (K.P.); p.deevanhxay@nuol.edu.la (P.D.)

**Keywords:** sodium alginate, calcium alginate, biocomposites, thermo-compression, reinforcement

## Abstract

Alginate is a biocompatible and biodegradable polymer derived from seaweed. It has been extensively researched and developed for various applications. However, its poor mechanical properties present a significant drawback that limits its use in multiple fields. Furthermore, the fabrication of reinforced alginate films using conventional melt processing has the potential for scaling up production. This study aimed to enhance the mechanical properties of sodium alginate (SA) films by incorporating calcium alginate (CA) powder. The SA/CA biocomposite films were created using a thermo-compression technique, with glycerol acting as a plasticizer for the SA matrix. Various CA contents—2.5, 5, 10, and 20 wt%—were investigated. Scanning electron microscopy and energy dispersive spectroscopy revealed good interfacial adhesion between the SA film matrix and the CA powder. As the CA content increased, the moisture content of SA/CA biocomposite films decreased. The addition of CA powder significantly improved the tensile properties of the SA films. Based on the tensile test, SA/CA biocomposite films with 20 wt% CA powder exhibited a maximum tensile strength of 11.7 MPa and a Young’s modulus of 234.7 MPa. These results indicate a substantial increase of 208% in maximum tensile strength and 907% in Young’s modulus compared to SA films without CA. These findings indicated that the CA powder serves as an effective reinforcing filler for thermo-compressed SA films, which could lead to the development of high-strength alginate-based products for potential use in various applications, including biomedical, agricultural, and packaging applications.

## 1. Introduction

Alginate is an abundant polysaccharide sourced from seaweed, which is approved by the United States Food and Drug Administration [1,2]. Because of its thickening capabilities, cost-effectiveness, biocompatibility, renewability, biodegradability, and favorable film-forming property [1,3,4], alginate has been widely researched for applications in various fields, including wastewater treatment [5,6], biomedical [7,8,9,10], agricultural [11,12], and packaging [13,14,15]. Alginate is generally available in the form of sodium alginate (SA), which is water-soluble and suitable for the forming and various applications mentioned above.

Most alginate-based products are derived from alginate aqueous solutions through evaporation processes. These processes include techniques such as solution casting, coating, wet spinning, and 3D printing [1,3,4,16]. The current formulation method restricts the scalability of alginate-based products for industrial production [4], which poses challenges for meeting the growing demand in sectors such as biomedical, packaging, and agriculture. Consequently, research is being conducted on the formation of alginate-based films using thermomechanical techniques, but there is still limited reporting [17,18,19,20]. Gao et al. [17,18] used thermomechanical processes to prepare plasticized SA films. They found that glycerol acted as a more effective nonvolatile plasticizer than sorbitol, and the prepared films showed decreasing strength and increasing flexibility as the plasticizer content increased. Chen et al. [19,20] also used glycerol as a plasticizer in the preparation of SA films using thermomechanical processes. Therefore, further research is needed on the preparation of alginate-based products using thermomechanical processes for clearer knowledge, especially for reinforced alginate products.

Alginate’s poor mechanical properties significantly limit its applications in various fields [1,21]. Therefore, research on the reinforcement of alginate-based products is of great importance to expand their application range and achieve greater efficiency. There are many studies reporting enhancements to the strength of alginate-based products, including cross-linking, addition of nanoparticles (nanocellulose, nanoclays, hydroxyapatite, silica, and graphene), and plant extracts (Aloe vera extract, peanut red skin extract, and *Clitoria ternatea* extract) [3,4]. These reinforced alginate films are prepared using the solvent casting technique, which has significant limitations in effectively blending with fillers and in scaling up to industrial levels due to the potential for poor filler dispersion and the use of a large amount of solvent, which requires a long evaporation time [4,22]. The use of calcium alginate powder as a discrete particulate filler in thermo-compressed alginate films has not been reported.

Calcium alginate (CA) features a cross-linked structure of alginate, which provides enhanced mechanical properties and thermal stability compared to SA. This improvement occurs because calcium ions create cross-links between alginate chains, leading to a stronger and more stable gel structure [23]. It is widely recognized that the crosslinking of alginate with calcium ions results in the formation of the egg-box structure [1,3,4,24]. This crosslinking enhances the strength and water resistance of alginate-based products, making them suitable for various biomedical applications. These applications include wound dressings, tissue engineering scaffolds, cell encapsulation, drug delivery, and artificial organ construction [25]. Crosslinking with calcium ions improves the properties of alginate films, according to reports [26,27]. Rhim [26] reported that alginate films showed an increase in tensile strength from 33.6 MPa to 85.9 MPa, a decrease in elongation at break from 14.0% to 3.8%, and a decrease in water solubility from close to 100% to 16.7% when SA films were immersed in a 5% *w*/*v* calcium chloride solution for 5 min. These CA films were prepared using the solvent casting technique of SA solution before Ca^2+^-crosslinking.

However, there are no reports of alginate products reinforced with non-toxic and biodegradable CA powder. Since CA has better mechanical and thermal properties compared to SA, it is expected to reinforce the SA matrix. Furthermore, the CA powder is anticipated to maintain its particle form during thermomechanical processing due to its cross-linked structures. In addition, previous research has not yet prepared reinforced SA films using thermomechanical processes [17,18,19,20]. Therefore, it is anticipated that it will be possible to prepare fully biocompatible and biodegradable CA-reinforced alginate products using thermomechanical techniques with the potential for scaling up production and creating new biocomposite materials for specific applications in various fields, including biomedical, agricultural, and packaging.

In this study, we developed SA/CA biocomposite films with varying CA contents using the thermomechanical technique. The CA powder was produced by initially cross-linking SA powder with calcium ions before combining it with the SA powder. This mixture of SA and CA powders was then plasticized with a glycerol solution before undergoing the thermomechanical process. We evaluated the impact of CA content on several properties of the thermo-compressed SA/CA biocomposite films, including chemical functional groups, phase morphology, crystalline structures, thermal stability, moisture content, film opacity, and mechanical properties.

## 2. Results and Discussion

### 2.1. Characterization of SA and CA Powders

#### 2.1.1. Water Solubility

The water solubility values for the SA and CA powders were found to be 100 ± 0% and 3.25 ± 0.56%, respectively. The crosslinking conditions employed in this study led to the nearly complete crosslinking of the SA powder, which rendered the CA powder effectively insoluble. After the water solubility test, the CA powder retained its fine powder consistency and showed no signs of aggregation.

#### 2.1.2. ATR-FTIR Analysis

**Figure 1:**
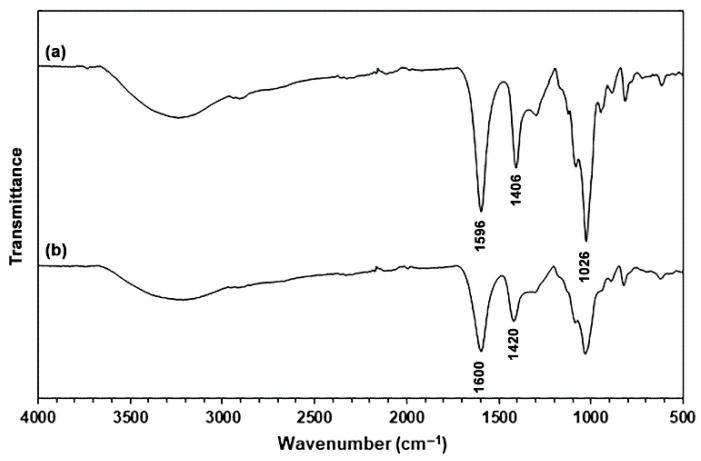
ATR-FTIR spectra of (**a**) SA and (**b**) CA powders.

Figure 1 shows ATR-FTIR spectra of SA and CA powders. The ATR-FTIR spectrum of the SA powder exhibited the absorption bands for the stretching vibration of hydroxyl (-OH) groups in the range of 3000–3700 cm^−1^, asymmetric and symmetric stretching vibrations for the carboxylate (-COO^−^) groups at 1596 cm^−1^ and 1406 cm^−1^, respectively [28], and stretching vibration for the C-O-C bonds at 1026 cm^−1^ [18,21]. The CA powder has an ATR-FTIR spectrum that is the same pattern as the SA powder. However, the absorption bands corresponding to the asymmetric and symmetric stretching vibration for the -COO^−^ groups in CA powder shifted to higher wavenumbers at 1600 cm^−1^ and 1420 cm^−1^, respectively. These findings indicated that they are characteristic of CA powder [18,27].

#### 2.1.3. Thermal Decomposition Properties

**Figure 2:**
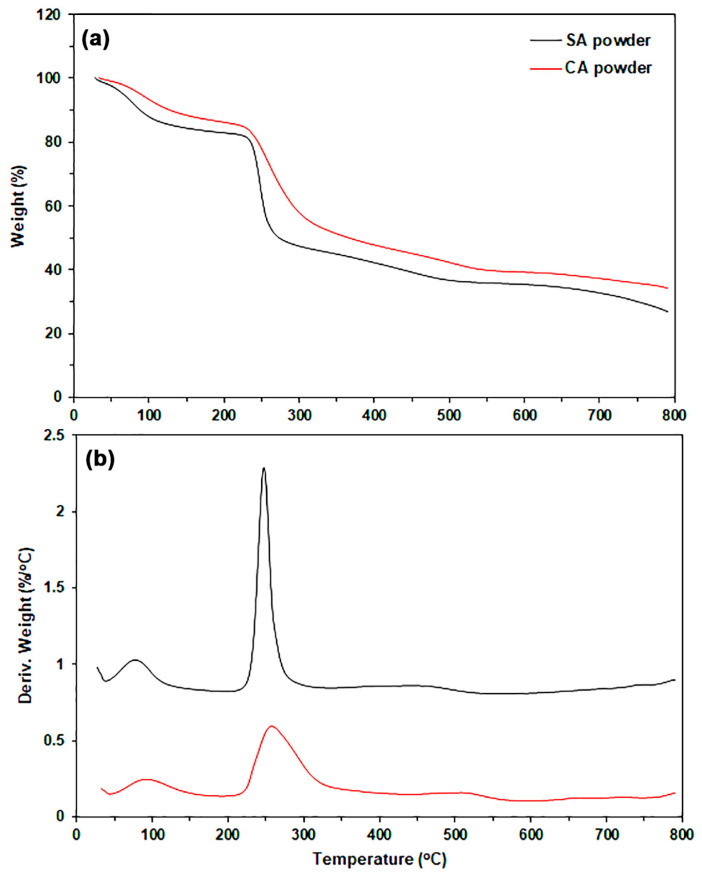
(**a**) TG and (**b**) DTG thermograms of SA and CA powders.

The thermal decomposition properties of SA and CA powders were evaluated using thermogravimetric (TG) and derivative TG (DTG) thermograms, as illustrated in Figure 2. Table 1 summarizes the TG and DTG results. The TG thermograms of both SA and CA powders exhibited two main stages of thermal decomposition. The first stage is in the range of 50–150 °C, which was attributed to the evaporation of bound water, and the second stage is in the range of 200–350 °C, which was assigned to the decomposition of alginate chains [18,21,29].

The decomposition temperatures at weight losses of 5% (T_5%_), 10% (T_10%_), and 50% (T_50%_) obtained from the TG thermogram, along with the decomposition temperature at maximum rate (T_max_) derived from the DTG thermogram, were all higher for CA powder than for SA powder. Weight losses at T_5%_ and T_10%_ are attributed to the evaporation of moisture from the sample powders. CA powder exhibits lower moisture absorption compared to SA powder, likely due to the crosslinked “egg box” structure of CA powder. The weight loss observed at T_50%_ and T_max_ is believed to result from the thermal decomposition of alginate chains [30,31]. The crosslinked structure of CA powder contributes to higher thermal decomposition temperatures, indicating greater thermal stability, compared to SA powder.

#### 2.1.4. Elemental Analysis

**Figure 3:**
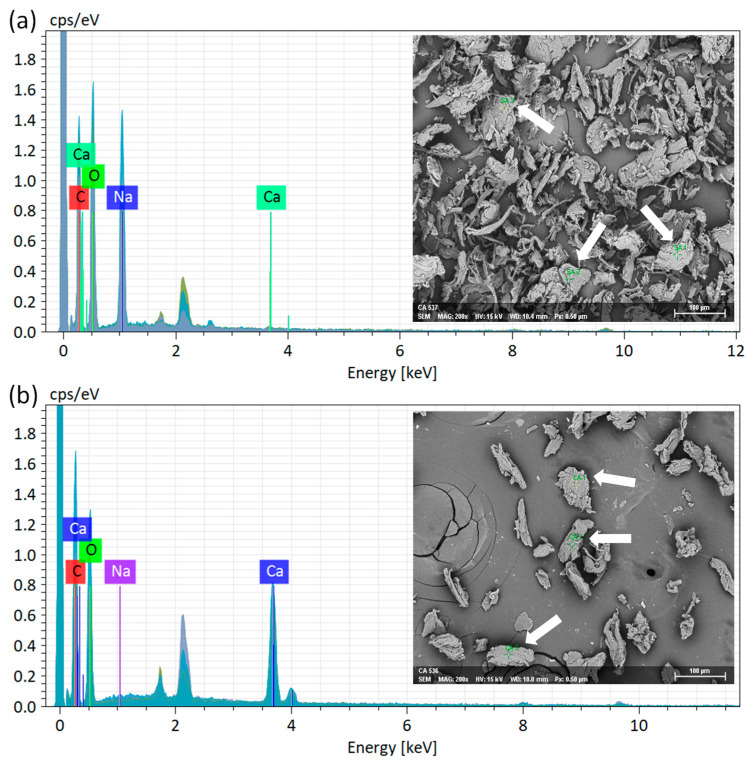
EDS spectra of (**a**) SA and (**b**) CA powders (white arrows indicate points for elemental analysis).

Figure 3a and Figure 3b display the EDS spectra for SA and CA powders, respectively, acquired via point analysis. The CA particles maintain their irregular shapes, much like the SA particles. Consequently, the CA powder preparation process in this study did not alter the particle shape. This suggests that rapid crosslinking by Ca^2+^ ions occurs on the surface of the SA particles, thereby preventing any changes in particle shape. Table 2 presents a summary of elemental compositions. The SA powder exhibited contents of Na and Ca at 13.23 wt% and 0.17 wt%, respectively. Conversely, the CA powder comprised 0.25 wt% of Na and 14.55 wt% of Ca. The EDS findings clearly indicate that Na and Ca are the dominant elements in SA and CA powders, respectively. This observation confirms the successful crosslinking of alginate with calcium ions, resulting in the formation of CA powder.

#### 2.1.5. Crystalline Structures

**Figure 4:**
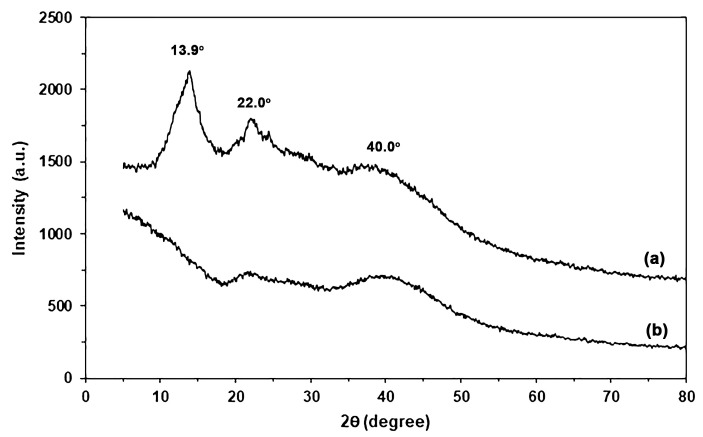
XRD profiles of (**a**) SA and (**b**) CA powders.

Figure 4 shows XRD profiles of SA and CA powders. The SA powder in Figure 4a exhibited XRD broad peaks at 2θ = 13.9° and 22.0°, assigned to two separate amorphous regions [17,18]. For CA powder, the XRD peak at 2θ = 13.9° disappeared, and the intensity of the XRD peak at 2θ = 22.0° decreased, as shown in Figure 4b. This finding may be explained by Ca^2+^-crosslinking, which induces alginate chains closer together, resulting in a larger area of an amorphous region with shorter distances between alginate chains [17]. A broad XRD peak at 2θ = 40.0° was assigned to the cross-linked structure of alginate with calcium ions [21]. The small peak at 2θ = 40.0° for SA powder can be explained by residue calcium ions, as informed by the supplier (0.3 wt% calcium content). The intensity of this broad XRD peak at 2θ = 40.0° increased when SA powder was cross-linked with calcium ions. These findings support that the alginate was cross-linked with calcium ions.

### 2.2. Characterization of SA/CA Biocomposite Films

#### 2.2.1. ATR-FTIR Analysis

**Figure 5:**
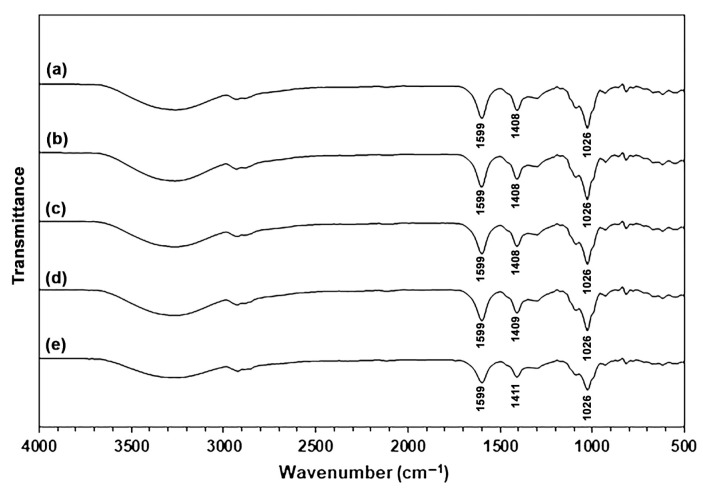
ATR-FTIR spectra of (**a**) SA film and SA/CA biocomposite films with CA contents of (**b**) 2.5, (**c**) 5, (**d**) 10, and (**e**) 20 wt%.

Figure 5 shows ATR-FTIR spectra of SA and SA/CA biocomposite films. The ATR-FTIR spectrum of the SA film exhibited the absorption bands for the stretching vibration of -OH groups in the range of 3000–3700 cm^−1^, the stretching vibration of C-H bonds in the range of 2800–3000 cm^−1^, the asymmetric and symmetric stretching vibration for the -COO^−^ groups at 1599 cm^−1^ and 1408 cm^−1^, respectively, and the stretching vibration for the C-O-C bonds at 1026 cm^−1^ [18,21]. Compared to SA powder in Figure 1a, glycerol-plasticized SA film in Figure 5a showed a higher intensity of the absorption bands for -OH groups and the C-H bonds as provided by glycerol molecules [18,32]. All the SA/CA biocomposite films have ATR-FTIR spectra that have the same pattern as the glycerol-plasticized SA film.

The band of the symmetric stretching vibration for the -COO^−^ groups in SA films without CA is observed at 1408 cm^−1^, which is slightly higher than the wavenumber of 1406 cm^−1^ for SA powder. This difference may result from the mixing of SA at 60 °C in an internal mixer, followed by thermo-compression at 120 °C. This process could lead to enhanced crosslinking in the SA matrix due to the presence of calcium ions in the SA powder. The symmetric -COO^−^ bands were also detected at a wavenumber of 1408 cm^−1^ in the SA/CA films containing 2.5 and 5 wt% of CA. The bands of the symmetric -COO^−^ groups for SA/10% CA and SA/20% CA films were observed at 1409 cm^−1^ and 1411 cm^−1^, respectively. The results indicate that Ca^2+^-crosslinking may transpire between the -COO^−^ groups of the SA matrix and the Ca^2+^ ions, likely at the surface of the CA particles [27]. But for SA/2.5% CA and SA/5% CA films that did not show this change, the amount of Ca^2+^-crosslinking at the interface was too small to be detected by FTIR analysis.

#### 2.2.2. Thermal Decomposition Properties

**Figure 6:**
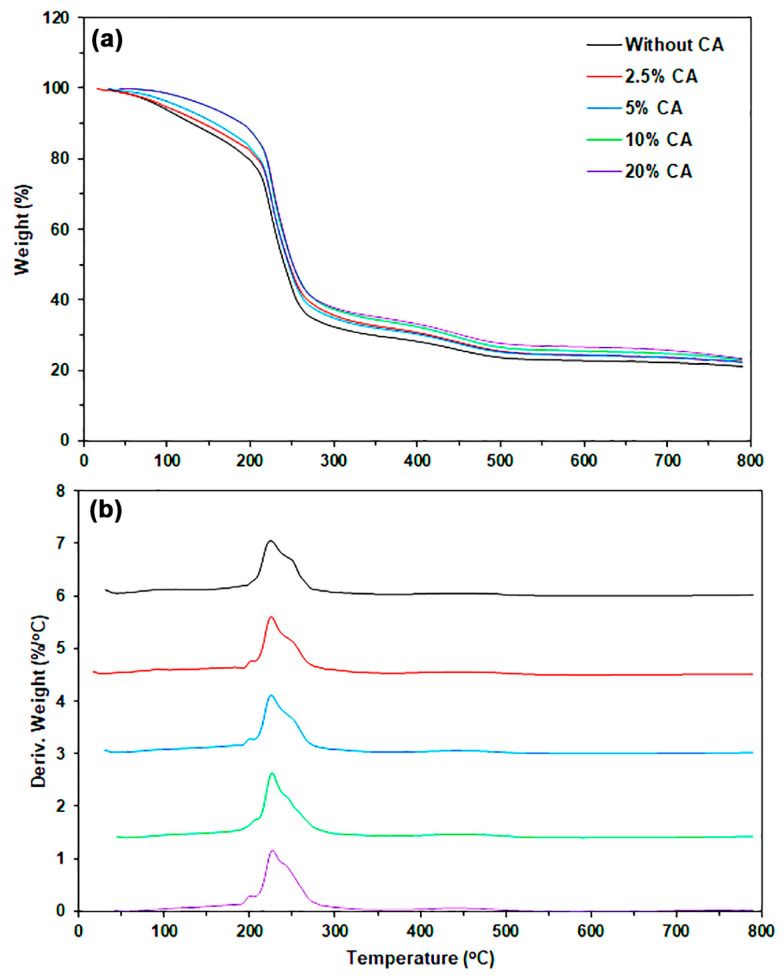
(**a**) TG and (**b**) DTG thermograms of SA and SA/CA biocomposite films.

The thermal decomposition properties of the film samples were determined using TG and DTG thermograms, as shown in Figure 6. Table 3 summarizes the results of thermal decomposition properties. The values of T_5%_, T_10%_, and T_50%_ were found to increase with the increase in CA content. The increase in T_5%_ and T_10%_ values indicated that absorbed water decreased with an increase in CA content. This result may be explained by CA powder having higher moisture resistance than the SA film matrix [26,27].

This study presents the T_max_ values for SA powder, CA powder, and glycerol-plasticized SA film, as detailed in Table 1 and Table 3, which are 248 °C, 258 °C, and 225 °C, respectively. Therefore, the order of thermal stability is as follows: CA powder > SA powder > glycerol-plasticized SA film. Previous literature [18] indicates that glycerol-plasticized SA film produced via thermo-compression exhibits lower thermal stability in comparison to SA powder. The higher content of CA powder likely slows the rate of thermal decomposition in SA/CA biocomposites. This phenomenon explains the increase in the T_50%_ value following the addition of CA powder. The change occurs because the thermal stability of CA powder is significantly better than that of the SA film matrix. The SA film matrix, which undergoes thermal decomposition at T_max_, showed no significant change with increasing CA content, remaining in the range of 225–227 °C. TGA results also indicated that neither the SA matrix nor the CA powder exhibited thermal decomposition during film forming using the thermo-compression technique at 120 °C.

#### 2.2.3. Crystalline Structures

**Figure 7:**
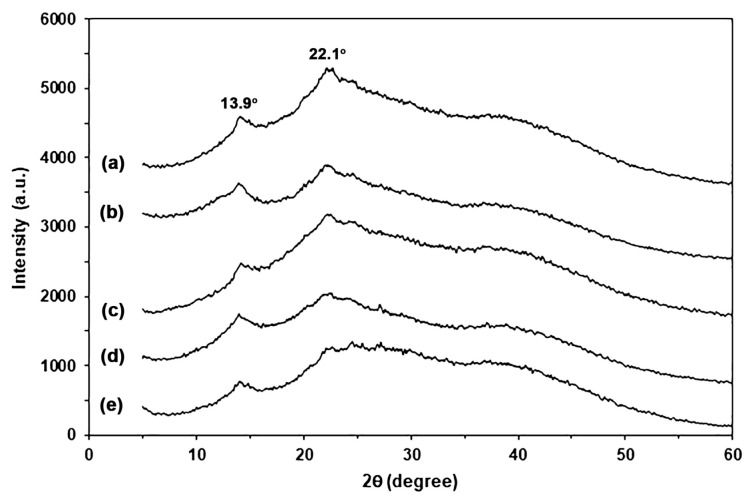
XRD profiles of (**a**) SA film and SA/CA biocomposite films with CA contents of (**b**) 2.5, (**c**) 5, (**d**) 10, and (**e**) 20 wt%.

Figure 7 shows XRD profiles of SA and SA/CA biocomposite films. The SA film showed a small XRD peak at 2θ = 13.9° and a broad XRD peak at 2θ = 22.1°, suggesting that the plasticization of glycerol induced the rearrangement of alginate chains in the second amorphous region at 2θ = 22.1° [17,18,33]. All the SA/CA biocomposite films have XRD profiles that have the same pattern as the glycerol-plasticized SA film. This finding suggests the addition of CA powder did not affect the crystalline structure of the glycerol-plasticized SA matrix. In addition, the intensity of XRD peaks at 2θ = 13.9° and 22.1° tended to decrease, and the intensity of broad XRD peaks at 2θ = 40.0° tended to increase as the CA content increased. CA powder had a broad XRD peak at 2θ = 40.0° due to the Ca^2+^-crosslinked structure [21]. Therefore, the changes in intensity of these XRD peaks are consistent with the decreasing SA content and increasing CA content of biocomposite films.

#### 2.2.4. Phase Morphology

**Figure 8:**
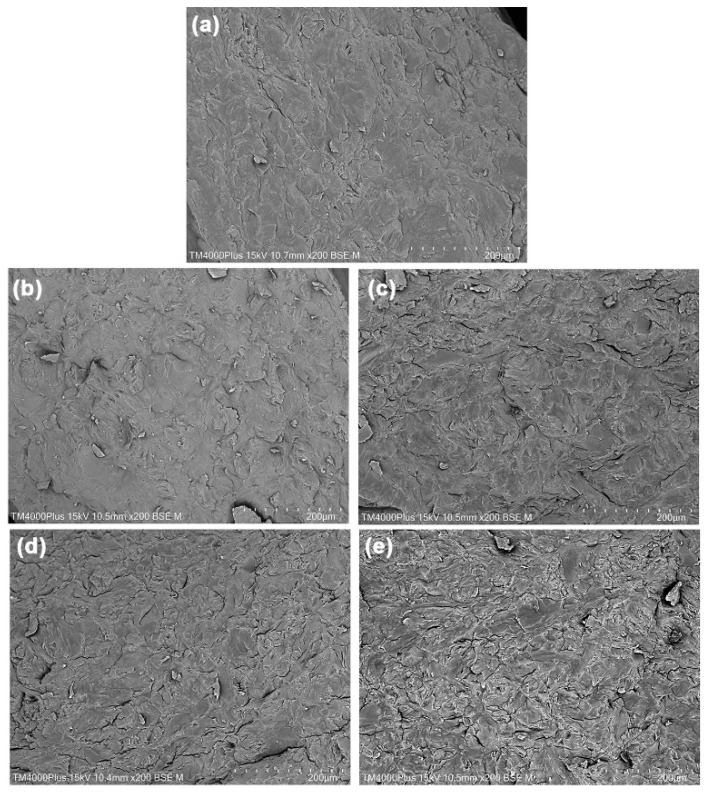
SEM images of cryo-fractured surfaces of (**a**) SA film and SA/CA biocomposite films with CA contents of (**b**) 2.5, (**c**) 5, (**d**) 10, and (**e**) 20 wt%. All bar scales = 200 µm.

The phase morphology of cryo-fractured surfaces of SA/CA biocomposite films was determined compared to the SA film using SEM images, as shown in Figure 8. Water and glycerol are utilized as plasticizers for alginate, breaking the hydrogen bonds between alginate molecules and allowing them chain mobility, which reduces their processing temperature [18]. The use of both water and glycerol results in efficient alginate melt molding. Although water is highly effective as a plasticizer for alginate, it is volatile and evaporates during the thermomechanical process. Glycerol, on the other hand, is a nonvolatile plasticizer that remains in the SA matrix after thermomechanical processing, resulting in flexible alginate films [17,18]. Mixing the SA powder with an aqueous glycerol solution at 60 °C is a pre-mixture preparation step, which is expected to cause the following significant structural changes: 1. Glycerol penetration: Glycerol begins to penetrate the SA particles. The heat of 60 °C reduces the viscosity of the glycerol solution, allowing it to diffuse more quickly. 2. Particle swelling: SA particles swell as glycerol breaks the hydrogen bonds between the alginate chains, causing the previously compacted structure to expand. 3. Softening: Although the SA particles have not yet coalesced, this temperature is expected to soften them, transforming them from a dry powder into a dough-like, plasticized SA. At this stage, they are significantly softened; they are yet completely homogeneous. During the thermo-compression process, plasticized SA pastes are subjected to high shear forces and heat, inducing a mechanism called sintering or particle coalescence. Molecular chains on the surfaces of adjacent SA particles diffuse and entangle, eliminating the boundaries between particles and forming a continuous film.

The glycerol-plasticized SA film without CA in Figure 8a showed some small SA particles in the film matrix. This finding indicates that the SA particles did not completely plasticize. The degree of plasticizing biopolymers may increase by increasing the temperature, shear force, and content of the plasticizer [18]. The cryo-fractured surfaces of SA/CA biocomposite films were rougher than the SA film. The surface roughness increased as the CA powder content increased. This effect was because the amount of embedded CA particles increased, as can be observed in SEM images of SA/CA films, as shown in Figure 8b–e. These findings suggest that CA powder remains a particle in the SA film matrix. A test was conducted to assess the stability of CA powder during thermomechanical processes by mixing 20 g of CA powder with 20 g of a 50 wt% aqueous glycerol solution in an internal mixer. The mixture underwent thermo-compression under conditions identical to those utilized for preparing SA/CA films. The resulting thermo-compressed CA appeared opaque and exhibited brittle characteristics, which complicated handling. Figure S1 presents SEM images of both the CA powder and the fractured surface of thermo-compressed CA. The CA particles in the thermo-compressed CA in Figure S1b retained a distinct morphology without coalescing. The characteristics of these CA particles closely resemble those of the CA powder depicted in Figure S1a. This observation confirms that CA powder maintains its particle structure throughout thermomechanical processes. Consequently, it can be inferred that CA powder within SA/CA film matrices also retains its particle structure.

Furthermore, the changes in the Ca^2+^-crosslinking, or egg-box structure, of CA powder during thermomechanical processes can also be investigated. The properties of thermo-compressed CA are illustrated. Figure S2 presents an ATR-FTIR spectrum of thermo-compressed CA. The symmetric stretching vibration band for the -COO^−^ groups in thermo-compressed CA is observed at 1418 cm^−1^, indicating the presence of a Ca^2+^-crosslinked structure [18,27]. In comparison, the bands for SA and CA powders shown in Figure 1 appear at 1406 cm^−1^ and 1420 cm^−1^, respectively. This suggests that the egg-box structure of thermo-compressed CA is partially disrupted during the thermomechanical processes. Additionally, the XRD profile of thermo-compressed CA, depicted in Figure S3, reveals two distinct amorphous regions of alginate, represented by broad XRD peaks at 2θ = 14.0° and 22.0° [17,18], along with the egg-box structure of alginate indicated by a broad XRD peak at 2θ = 40.0° [21]. The intensities of these XRD broad peaks of thermo-compressed CA, which represent two distinct amorphous regions of alginate, were found to be higher than the intensity of a broad XRD peak at 2θ = 40.0°, indicative of an egg-box structure, when compared to those of CA powder in Figure 4b. However, these XRD peak intensities reveal two distinct amorphous regions of alginate, remaining lower than those observed for SA powder in Figure 4a. The results suggest that some of the Ca^2+^-crosslinked structures in CA powder within SA/CA films may have been compromised during the thermomechanical processes. However, the CA powder still retains an egg-box structure, which allows it to maintain a particle-like form without undergoing sufficient plasticization with water and glycerol to cause the coalescing of CA particles during thermomechanical properties, as illustrated in Figure S1b.

All the SA/CA films exhibited a tightly packed structure. The SEM results indicate that there is good interfacial adhesion between dispersed CA particles and the SA matrix [34,35]. The FTIR analysis suggests that the observed effect is due to Ca^2+^-crosslinking occurring on the surface of CA particles and the SA matrix. Therefore, Ca^2+^-crosslinking at interfaces between these components is likely the main factor influencing interfacial adhesion. In addition, this interfacial adhesion may be caused by chain entanglements and hydrogen bonds that occur between the alginate chains of CA particles and those of the film matrix [36].

#### 2.2.5. Elemental Analysis

**Figure 9:**
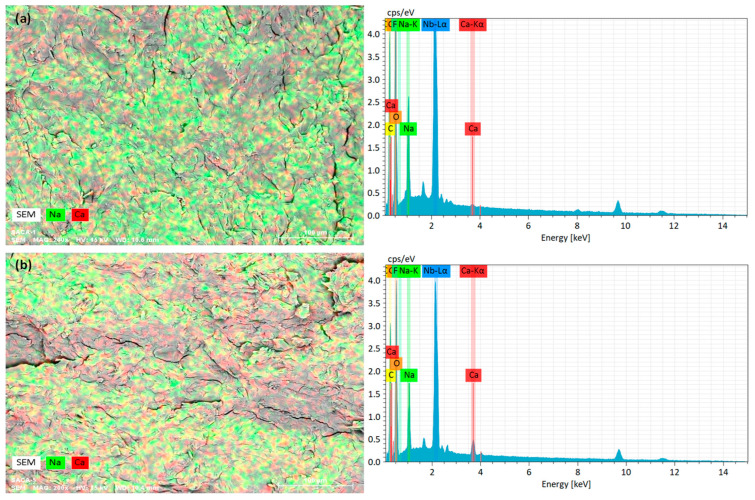
EDS elemental mapping and EDS spectra of (**a**) SA and (**b**) SA/20% CA biocomposite films.

The elemental analysis of the film cross-sections of SA/CA biocomposite films was determined by the EDS technique. Figure 9 shows examples of the ESD elemental mapping and the EDS spectra of SA and SA/20% CA biocomposite films. The green and red colors indicate Na and Ca elements, respectively. The elemental analysis results are summarized in Table 4. The SA film exhibits a predominantly green area of the Na element. However, a red-colored area was also observed for the SA film. This conclusion is because, according to the SA supplier’s data, the calcium content is approximately 0.3 wt%. The SA/20% CA biocomposite film was found to have a higher red area of the Ca element compared to the SA film. From Table 4, the content of the Ca element significantly increased as the CA content increased. This finding indicates that CA particles are well distributed in the SA film matrix.

#### 2.2.6. Moisture Content and Film Opacity

Table 5 summarizes the thickness, moisture content, and opacity of the film samples. The film thickness increased as the CA content increased. This increased film thickness may be explained by the dispersed CA particles in the biocomposites, which reduce the melt flow property of plasticized SA during the thermo-compression process. The moisture content of the film decreased as the CA content increased. A reason for this decrease in moisture content was that CA particles exhibited higher water resistance than the SA film matrix, according to the literature [26,27,29]. The crosslink structure of CA particles caused the alginate chains to be closer together, which reduced the diffusion of absorbed water molecules [26].

**Figure 10:**
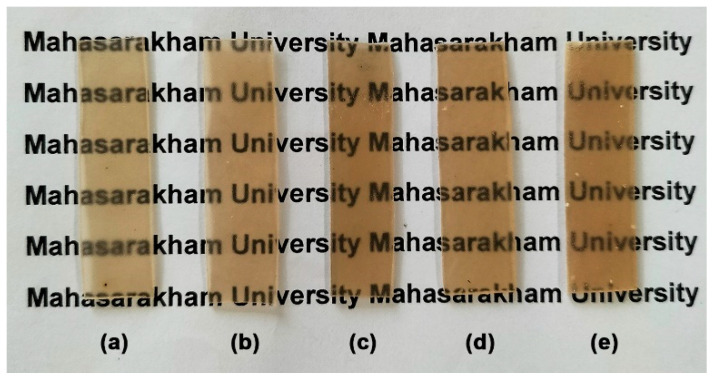
Visual transparency of (**a**) SA film and SA/CA biocomposite films with CA contents of (**b**) 2.5, (**c**) 5, (**d**) 10, and (**e**) 20 wt%.

The film opacity of the SA/CA films containing 2.5, 5, and 10 wt% CA was nearly identical to that of the SA film, which measured 0.759 ± 0.032 mm^−1^. This means that adding 2.5, 5, or 10 wt% CA did not change the film opacity of the SA film. However, when the CA content was 20 wt%, the film opacity significantly increased (0.814 ± 0.031 mm^−1^). Figure 10 illustrates the visual transparency of SA and SA/CA biocomposite films. All film samples exhibited a light-brown color, which is characteristic of alginate film [18]. It was clearly observed that when 20% CA powder is added, CA particles agglomerate, appearing as clusters of white dots scattered throughout the film matrix. However, all the films are still transparent enough to allow the letters underneath to be read.

#### 2.2.7. Mechanical Properties

**Figure 11:**
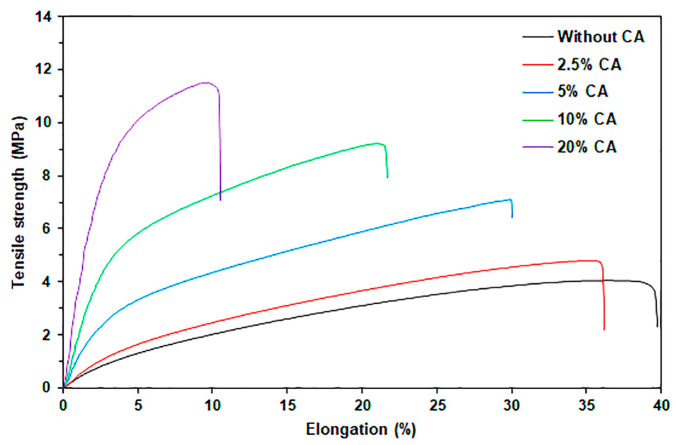
Tensile curves of SA and SA/CA biocomposite films with varying CA content.

Figure 11 displayed the tensile curves of the SA and SA/CA biocomposite films. Table 6 provides a summary of the tensile test results for the film samples. The SA film without CA exhibits the lowest maximum tensile strength (3.8 MPa) and Young’s modulus (23.3 MPa), indicating poor mechanical properties. The incorporation of CA powder enhances both the maximum tensile strength and Young’s modulus of the SA films. The maximum tensile strength and Young’s modulus of the SA/CA biocomposite films significantly increased as the CA content increased. Specifically, the maximum tensile strength increases by 24%, 97%, 140%, and 208% for the 2.5%, 5%, 10%, and 20% CA additions, respectively, compared to the SA film. It has been reported that the CA films exhibited higher tensile strength than the SA films [26,37,38]. Similarly, Young’s modulus increased by 24%, 134%, 467%, and 907% with the addition of 2.5%, 5%, 10%, and 20% CA, respectively, in comparison to SA film. The SEM analysis supports this finding, indicating good interfacial adhesion between the CA particles and the SA film matrix. The FTIR results for the SA/10% CA and SA/20% CA films indicate that interactions occur between them. Therefore, it is expected that effective stress transfer occurs from the SA matrix to stronger CA particles [4,39], leading to the conclusion that the CA powder is an effective reinforcing filler for SA film.

The significant increase in the Young’s modulus of SA/CA films can be explained by the “rule of mixture”, which relates to the volume fraction and Young’s modulus of fillers. Since CA has a higher Young’s modulus than SA [4,22], the Young’s modulus of SA/CA films increases as the proportion of CA powder rises. However, SA/CA films exhibit largely higher Young’s modulus values than SA films. This substantial increase is attributed to two main phenomena that are expected to be synergistic effects of the increase in Young’s modulus: First, CA particles may absorb glycerol, leading to a phenomenon known as anti-plasticization, which further enhances the film’s Young’s modulus [40]. Second, higher CA content in SA/CA films leads to lower moisture content, which also contributes to the increase in the film’s Young’s modulus [41].

From Figure 10, it is clear that aggregation of CA particles occurs in the SA/20% CA films; however, the maximum tensile strength of the SA/CA films continues to increase. Generally, filler aggregation is regarded as a defect that can compromise tensile strength. Despite the formation of large aggregates, if there is sufficient polymer wetting, the retained polymer can act as a “bridge” to transfer force, enabling the aggregate structure to serve as reinforcement rather than merely a defect [42]. The FTIR and SEM analyses of the SA/20% CA films reveal interface interactions and demonstrate satisfactory phase compatibility, respectively, between the CA particles and the SA matrix, indicating that there is adequate polymer wetting to enhance its tensile strength.

The elongation at break of the biocomposite films consistently decreased with increasing CA content. The observed reduction in elongation at break, attributed to the incorporation of CA, may result from interactions between CA particles and SA film matrix. Such interactions could restrict SA chain mobility within the film matrix, potentially resulting in reduced elasticity for the films [26,43,44]. Additionally, absorbed water molecules can induce plasticization, which increases the flexibility of the film matrix [45,46]. The reduced moisture content in the films may also explain why the SA/CA films exhibited lower values of elongation at break.

Table 7 compares the tensile properties of thermo-compressed SA films with previously reported research. The glycerol-plasticized SA films without filler prepared in this research exhibited mechanical properties similar to those previously reported [17,18]. With the addition of 20% CA powder, the SA/CA films showed higher maximum tensile strength (11.7 MPa) and Young’s modulus (234.7 MPa) than all other SA films and lower elongation at break (11.6%). This finding confirms that CA powder acts as a reinforcing filler for thermo-compressed SA films.

The various applications of alginate films require different levels of strength and flexibility. Therefore, a balance between strength and flexibility for specific applications is necessary. The commercial wound dressing Acticoat^®^ possesses a maximum tensile strength of 4.60 MPa and an elongation at break of 27.13% [47]. The SA/5% CA films produced in this study demonstrate a maximum tensile strength of 7.5 MPa and an elongation at break of 30.9%. The mechanical properties indicate that the SA/5% CA films possess adequate strength and flexibility for prospective use as a wound dressing. Xie et al. [48] formulated a chitosan–collagen–alginate (CCA) composite for wound dressing applications, attaining a maximum tensile strength of 0.36 MPa and an elongation at break of 4.96%. In vivo wound healing assessments revealed that this CCA composite displayed enhanced healing rates during the initial five days relative to sterilized gauze and chitosan dressings. Therefore, the SA/20% CA films prepared in this research, which have a higher maximum tensile strength (11.7 MPa) and elongation at break (11.6%) than the CCA composite, possess potential strength and flexibility for similar wound dressing applications.

## 3. Experimental

### 3.1. Materials

Sodium alginate (SA) powder, with a particle size of less than 170 mesh and a calcium content of less than 0.3 wt%, was purchased from Chanjao Longevity Co., Ltd. (Bangkok, Thailand). The viscosity of SA determined from a 1 wt% solution at 20 °C (Brookf. DV3T No. 62, 20 rpm) was 890 cps. The mannuronic acid/guluronic acid (M/G) ratio of SA powder is 60/40. Glycerol (QReC brand, 99.5%, AR grade) was obtained from Smart Science Co., Ltd. (Pathum Thani, Thailand). Calcium chloride (CaCl_2_, 97.0%) was purchased from Kemaus (Cherrybrook, NSW, Australia).

### 3.2. Preparation of Calcium Alginate Powder

The SA powder (1 g) was stirred in 500 mL of 10 wt% CaCl_2_ aqueous solution for 1 h [3]. After the formation of calcium alginate (CA) powder, it was centrifuged at 5000 rpm for 10 min and washed three times with deionized water. The powder was then dried in an air oven at 100 °C for 24 h. The resulting CA powder was stored at a temperature of 25 °C with a relative humidity (RH) of 50% for 14 days prior to use and characterization.

### 3.3. Preparation of SA/CA Biocomposite Films

The production of plasticized SA pellets commenced with the manual mixing of SA (20 g) and a glycerol aqueous solution (20 g). The aqueous glycerol solution was prepared by combining 10 g of glycerol with 10 g of distilled water. The resulting SA paste was then processed in an internal mixer (Polylab OS system, HAAKE, Waltham, MA, USA) at a temperature of 60 °C and a rotor speed of 60 rpm for a duration of 20 min. Films made from SA were produced through the thermo-compression of plasticized SA using an Auto CH Carver compression molding machine (Wabash, IN, USA). The procedure was carried out at 120 °C for 5 min with a compression force of 5 MPa [17,18]. The resulting SA film was cooled using cool plates for 5 min while maintaining a compression force of 5 MPa.

The SA/CA biocomposite films were produced under the same conditions. SA and CA powders were first combined in a dry mixture using a Moulinette Essential food chopper (MB520138, Tefal, Hangzhou, China) for 5 min before being kneaded with a glycerol aqueous solution. These pastes were then blended using an internal mixer before thermo-compression under the same conditions for preparing the SA films. The SA/CA biocomposite films with CA contents of 2.5, 5, 10, and 20 wt% relative to the weight of SA were examined. The SA and SA/CA biocomposite films were stored for 14 days at 25 °C and 50% RH prior to characterization. This storage period allowed for moisture balance, complete alignment of alginate molecules, and reduction in residue stress in the films [17,18].

### 3.4. Characterization of SA Powder, CA Powder, and SA/CA Biocomposite Films

#### 3.4.1. Water Solubility

The sample powder (0.2 g) was dried at 80 °C for 24 h before weighing (W_1_). The powders were kept in 50 mL of distilled water with constant stirring. After 24 h, the powders were separated by centrifuging at 5000 rpm for 10 min and dried at 80 °C for 24 h before weighing (W_2_). Then, the water solubility was calculated using Equation (1). The water solubility was averaged based on three measurements.

Water solubility = [(W_1_ − W_2_)/W_1_] × 100% (1)

#### 3.4.2. Fourier Transform Infrared Spectroscopy (FTIR)

The FTIR analysis was carried out using a Bruker Invenio-S FTIR spectrometer (Karlsruhe, Germany) in an attenuated total reflection (ATR) module to determine the chemical functional groups. The samples were analyzed in the transmission mode in the 500–4000 cm^−1^ range with an average scan of 32 and 4 cm^−1^ resolution.

#### 3.4.3. Thermal Decomposition

The thermal decomposition of samples was evaluated using a thermogravimetric analyzer (TGA, SDT Q600, TA-Instruments, New Castle, DE, USA). The samples were heated under nitrogen flow from 50 °C to 800 °C. The heating rate of 20 °C.min^−1^ and nitrogen flow rate of 20 mL.min^−1^ were used.

#### 3.4.4. Crystalline Structures

The crystalline structures of samples were determined using an X-ray diffractometer (XRD D8 Advance, Bruker, Karlsruhe, Germany). A CuKα source was operated at 40 kV and 40 mA. The samples were scanned from 2θ = 5° to 60° with a scan mode at 2° min^−1^ and a 0.02° step.

#### 3.4.5. Film Morphology

The film morphology of cryo-fractured surfaces of film samples was analyzed using a scanning electron microscope (SEM, TM4000Plus, Hitachi, Tokyo, Japan) at 15 kV under vacuum conditions. The samples were sputter coated with a thin layer of gold before the SEM analysis.

#### 3.4.6. Elemental Analysis

The elemental analysis of samples was determined using an energy dispersive X-ray spectroscope (EDS, Bruker, Karlsruhe, Germany) with the XFlash detector series. The film samples were cryo-fractured in liquid nitrogen before EDS scans. The elemental compositions of powder and film cross-section samples were determined by point and mapping analyses, respectively.

#### 3.4.7. Moisture Content

The sample film (20 × 20 mm) was weighed (W_i_) before drying it at 105 °C for 24 h. The dried film was kept in a desiccator before weighing (W_f_). Then, the moisture content was calculated using Equation (2). The moisture content was averaged based on three determinations.

Moisture content = [(W_i_ − W_f_)/W_i_] × 100% (2)

#### 3.4.8. Film Opacity

The film opacity of film samples was evaluated using an ultraviolet–visible spectrophotometer (Cary 60, Agilent Technologies, Victoria, Australia). For this purpose, the absorbance of film samples at a wavelength of 600 nm (A_600_) was measured. Then, the film opacity was calculated according to Equation (3) [49]. The film opacity was averaged using three measurements.

Film opacity (mm^−1^) = A_600_/X (3)

where X is the thickness of the film sample (mm).

#### 3.4.9. Mechanical Properties

Tensile test was performed to determine the mechanical properties of the films (tensile strength, elongation, and Young’s modulus) using a LY-1066B tensile tester (Dongguan Liyi Environmental Technology Co., Ltd., Dongguan, China). The initial gap was 40 mm and a strain rate of 50 mm.min^−1^. Five specimens of each sample were tested and averaged.

### 3.5. Statistical Analysis

The results of tensile properties, moisture content, and film opacity were determined at least three times and presented as the mean ± standard deviation (SD). Differences between film samples were compared for statistical significance using the one-way ANOVA, followed by Duncan’s post hoc test. Mean property values with different letters were significantly separated (*p* < 0.05).

## 4. Conclusions

The glycerol-plasticized sodium alginate (SA) films reinforced with calcium alginate (CA) powder were successfully fabricated using the internal mixing followed by the thermo-compression technique. The effect of CA content on the properties of SA/CA biocomposite films was evaluated. The SA powder was cross-linked with calcium ions to produce CA powder, which was then characterized using FTIR, TGA, XRD, and EDS analyses. The results from these analyses indicated the characteristics of the CA powder. The SEM and EDS analyses demonstrated good interfacial adhesion between the SA film matrix and CA particles. As the CA content increased, the film thickness of SA/CA biocomposite films increased, while the moisture content decreased. Film opacity remained unchanged with varying CA content, except for the addition of 20% CA. Furthermore, both the maximum tensile strength and Young’s modulus of the SA/CA biocomposite films significantly increased as the CA content increased. The addition of 20% CA to SA/CA biocomposite films resulted in a maximum tensile strength of 11.7 MPa and a Young’s modulus of 234.7 MPa. This finding reflects a 208% increase in maximum tensile strength and a 907% increase in Young’s modulus compared to the SA films. This study illustrates that CA powder serves as an effective reinforcing agent, augmenting the mechanical properties of thermo-compressed SA films and rendering them more appropriate for diverse applications. The described methods may enable the advancement of additional SA biocomposites via conventional processing for potential use in various applications, including medical uses like wound dressings, tissue engineering, and drug delivery; agricultural applications such as seeding bags and mulch films; and packaging purposes. Additional information concerning specific properties is required for each application.

## Figures and Tables

**Table 1:** Thermal decomposition properties of SA and CA powders.

Powder	T_5%_ (°C) ^1^	T_10%_ (°C) ^1^	T_50%_ (°C) ^1^	T_max_ (°C) ^2^
SA	66	88	273	248
CA	87	126	364	258

^1^ Obtained from TG thermograms. ^2^ Obtained from DTG thermograms.

**Table 2:** Elemental analysis of SA and CA powders.

Powder	C Element (wt%)	O Element (wt%)	Na Element (wt%)	Ca Element (wt%)
SA	41.20 ± 1.13 ^b^	45.33 ± 0.72 ^a^	13.23 ± 1.67 ^b^	0.25 ± 0.06 ^a^
CA	36.64 ± 1.91 ^a^	48.64 ± 0.19 ^b^	0.17 ± 0.06 ^a^	14.55 ± 1.78 ^b^

Values are expressed as mean ± standard deviation (*n* = 3). Column values represented by the letters (^a^ and ^b^) demonstrate significant differences (*p* < 0.05).

**Table 3:** Thermal decomposition properties of SA and SA/CA biocomposite films.

CA Content (wt%)	T_5%_ (°C) ^1^	T_10%_ (°C) ^1^	T_50%_ (°C) ^1^	T_max_ (°C) ^2^
-	91	130	241	225
2.5	96	144	246	225
5	96	158	246	225
10	148	190	251	226
20	148	190	251	227

^1^ Obtained from TG thermograms. ^2^ Obtained from DTG thermograms.

**Table 4:** Elemental analysis of SA and SA/CA biocomposite films.

CA Content (wt%)	C Element (wt%)	O Element (wt%)	Na Element (wt%)	Ca Element (wt%)
-	37.12 ± 1.42 ^b^	51.98 ± 1.92 ^a^	10.61 ± 0.23 ^e^	0.29 ± 0.03 ^a^
2.5	37.17 ± 1.63 ^b^	52.16 ± 1.25 ^a^	10.03 ± 0.18 ^d^	0.64 ± 0.04 ^b^
5	36.65 ± 0.98 ^a,b^	52.79 ± 1.36 ^a^	9.58 ± 0.15 ^c^	0.98 ± 0.03 ^c^
10	35.75 ± 1.47 ^a^	53.35 ± 1.84 ^a,b^	9.04 ± 0.21 ^b^	1.86 ± 0.05 ^d^
20	34.15 ± 1.37 ^a^	54.41 ± 2.07 ^b^	8.61 ± 0.19 ^a^	2.83 ± 0.05 ^e^

Values are expressed as mean ± standard deviation (*n* = 3). Column values represented by the letters (^a^, ^b^, ^c^, ^d^, and ^e^ demonstrate significant differences (*p* < 0.05).

**Table 5:** Film thickness, moisture content, and film opacity of SA and SA/CA biocomposite films.

CA Content (wt%)	Film Thickness (mm)	Moisture Content (%)	Film Opacity (mm^−1^)
-	0.519 ± 0.011 ^a^	21.1 ± 1.4 ^d^	0.759 ± 0.032 ^a^
2.5	0.563 ± 0.008 ^b^	17.9 ± 2.1 ^c^	0.765 ± 0.028 ^a^
5	0.621 ± 0.014 ^c^	17.5 ± 1.6 ^b,c^	0.764 ± 0.035 ^a^
10	0.626 ± 0.010 ^c^	16.8 ± 0.8 ^a,b^	0.789 ± 0.022 ^a,b^
20	0.670 ± 0.015 ^d^	15.5 ± 1.1 ^a^	0.814 ± 0.031 ^b^

Values are expressed as mean ± standard deviation (*n* = 3). Column values represented by the letters (^a^, ^b^, ^c^, and ^d^) demonstrate significant differences (*p* < 0.05).

**Table 6:** Tensile properties of SA and SA/CA biocomposite films.

CA Content (wt%)	Maximum Tensile Strength (MPa)	Elongation at Break (%)	Young’s Modulus (MPa)
-	3.8 ± 0.5 ^a^	39.2 ± 1.8 ^e^	23.3 ± 2.4 ^a^
2.5	4.7 ± 0.4 ^a^	36.1 ± 2.4 ^d^	29.0 ± 3.2 ^b^
5	7.5 ± 0.6 ^b^	30.9 ± 2.5 ^c^	54.4 ± 5.1 ^c^
10	9.1 ± 0.8 ^c^	21.1 ± 1.9 ^b^	132.2 ± 10.6 ^d^
20	11.7 ± 1.1 ^d^	11.6 ± 2.1 ^a^	234.7 ± 12.5 ^e^

Values are expressed as mean ± standard deviation (*n* = 3). Column values represented by the letters (^a^, ^b^, ^c^, ^d^, and ^e^) demonstrate significant differences (*p* < 0.05).

**Table 7:** Comparative summary of tensile properties of thermo-compressed SA films.

Glycerol Content (wt%)	Filler	Maximum Tensile Strength (MPa)	Elongation at Break (%)	Young’s Modulus (MPa)	References
30	-	7 ± 1	54 ± 1	17 ± 3	[17]
30	-	3.0 ± 0.1	21 ± 8	15 ± 4	[18]
33	-	3.8 ± 0.5	39.2 ± 1.8	23.3 ± 2.4	This study
33	20% CA powder	11.7 ± 1.1	11.6 ± 2.1	234.7 ± 12.5	This study

## Data Availability

The raw data supporting the conclusions of this article will be made available by the authors on request.

## Supplementary Materials

The following supporting information can be downloaded at: https://www.mdpi.com/article/10.3390/md24040142/s1, Figure S1. SEM images of (a) CA powder and (b) fractured surface of thermo-compressed CA. All bar scales = 100 µm; Figure S2. ATR-FTIR spectrum of thermo-compressed CA; Figure S3. XRD profile of thermo-compressed CA.

## References

[1] Zhang X., Wang X., Fan W., Liu Y., Wang Q., Weng L. (2022). Fabrication, property and application of calcium alginate fiber: A review. Polymers.

[2] Cotas J., Lourenço M., Figueirinha A., Valado A., Pereira L. (2025). Seaweed polysaccharides: A rational approach for food safety studies. Mar. Drugs.

[3] Zdiri K., Cayla A., Elamri A., Erard A., Salaun F. (2022). Alginate-based bio-composites and their potential applications. J. Funct. Biomater..

[4] Xie F., Gao C., Avérous L. (2024). Alginate-based materials: Enhancing properties through multiphase formulation design and processing innovation. Mater. Sci. Eng. R Rep..

[5] Cai Z., Zhu C., Xiong P., Guo J., Zhao K. (2018). Calcium alginate-coated electrospun polyhydroxybutyrate/carbon nanotubes composite nanofibers as nanofiltration membrane for dye removal. J. Mater. Sci..

[6] Zhao X., Wang X., Lou T. (2021). Preparation of fibrous chitosan/sodium alginate composite foams for the adsorption of cationic and anionic dyes. J. Hazard. Mater..

[7] Diniz F.R., Maia R.C.A.P., Rannier Andrade L., Andrade L.N., Vinicius Chaud M., da Silva C.F., Corrêa C.B., de Albuquerque Junior R.L.C., Pereira da Costa L., Shin S.R. (2020). Silver nanoparticles-composing alginate/gelatin hydrogel improves wound healing in vivo. Nanomaterials.

[8] Dodero A., Scarfi S., Pozzolini M., Vicini S., Alloisio M., Castellano M. (2020). Alginate-based electrospun membranes containing ZnO nanoparticles as potential wound healing patches: Biological, mechanical, and physicochemical characterization. ACS Appl. Mater. Interfaces.

[9] Leu Alexa R., Ianchis R., Savu D., Temelie M., Trica B., Serafim A., Vlasceanu G.M., Alexandrescu E., Preda S., Iovu H. (2021). 3D printing of alginate-natural clay hydrogel-based nanocomposites. Gels.

[10] Kahya N., Kartun A., Korkut I.N., Usta C., Kuruca D.S., Gürarslan A. (2024). Silver nanowire-coated porous alginate films for wound dressing applications: Antibacterial activity, cell proliferation, and physical characterization. ACS Omega.

[11] Lv J., Chen P., Li S. (2025). Novel biodegradable mulch films made from vegetable stalk and sodium alginate. Inter. J. Biol. Macromol..

[12] Castro G.M., Tewelde D., Tubaldi E. (2025). Assessment of the use of sodium alginate for soil improvement in coastal applications. Sci. Rep..

[13] Ji Y., Zhao S., Yang Z., Chen Q., Wang X., Yang J. (2025). Intelligent film based on mulberry anthocyanin, sodium alginate, and gellan gum for monitoring pork freshness. LWT-Food Sci. Technol..

[14] Ning Q., Tang J., Chen H., Chen Q., Wu C., Pang J. (2025). Development and characterization of alginate/konjac glucomannan composite film reinforced with propolis extract and tea tree essential oil co-loaded Pickering emulsions for strawberry preservation. Ind. Crops Prod..

[15] Shah Y.A., Bhatia S., Al-Harrasi A., Tarahi M., Jawad M., Alam T., Dıblan S., Koca E., Aydemir L.Y., Thekkuden D.T. (2025). Extraction and applications of frankincense oleoresin as functional ingredient in pectin/sodium-alginate composite films for active packaging. LWT-Food Sci. Technol..

[16] Wang F., Lainé E., Lukova P., Katsarov P., Delattre C. (2026). Tailoring the properties of marine-based alginate hydrogels: A comparison of enzymatic (HRP) and visible-light (SPS/Ruth)-induced gelation. Mar. Drugs.

[17] Gao C., Pollet E., Avérous L. (2017). Innovative plasticized alginate obtained by thermo-mechanical mixing: Effect of different biobased polyols systems. Carbohyd. Polym..

[18] Gao C., Pollet E., Avérous L. (2017). Properties of glycerol-plasticized alginate films obtained by thermo-mechanical mixing. Food Hydrocoll..

[19] Chen P., Xie F., Tang F., McNally T. (2020). Unexpected plasticization effects on the structure and properties of polyelectrolyte complexed chitosan/alginate materials. ACS Appl. Polym. Mater..

[20] Chen P., Xie F., Tang F., McNally T. (2021). Graphene oxide enhanced ionic liquid plasticisation of chitosan/alginate bionanocomposites. Carbohyd. Polym..

[21] Abdel Aziz M.S., Salama H.E., Sabaa M.W. (2018). Biobased alginate/castor oil edible films for active food packaging. LWT-Food Sci. Technol..

[22] Manikandan G., Senthilkumar G., Chen C.-W., Nagarajan D., Chang J.-S., Dong C.-D. (2025). Valorization of brown seaweed into next-generation alginate bioplastic films: Functional enhancements and smart packaging applications. Carbohyd. Polym..

[23] Malektaj H., Drozdov A.D., deClaville Christiansen J. (2023). Mechanical properties of alginate hydrogels cross-linked with multivalent cations. Polymers.

[24] Wang H., Ren C., Bai W., Tang Z., Lei H., Tian H., Du G. (2025). Eucalyptus oil encapsulated within calcium-crosslinked sodium alginate for natural wood preservatives against fungi and termite. Ind. Crops Prod..

[25] Lee K.Y., Mooney D.J. (2012). Alginate: Properties and biomedical applications. Prog. Polym. Sci..

[26] Rhim J.-W. (2004). Physical and mechanical properties of water resistant sodium alginate films. LWT-Food Sci. Technol..

[27] Liu Z., Liu Q., Lin L., Wang Q., Ma W., Cheng Q., Yang J., Tang F., Xu M., Yang X. (2024). Stepwise reinforcement strategy for guar gum/sodium alginate based films: Introduction of carboxylated cellulose nanofibers by different methods and further calcium ion crosslinking. Food Hydrocoll..

[28] Mendes J.C., Valente J.F.A., Sousa F., Bernardino R., Bernardino S., Afonso C., Chagas B. (2026). Optimisation of alginate extraction and characterisation of polysaccharides from brown seaweed from the Portuguese Coast. Mar. Drugs.

[29] Weerapoprasit C., Prachayawarakorn J. (2016). Properties of biodegradable thermoplastic cassava starch/sodium alginate composites prepared from injection molding. Polym. Compos..

[30] Fajardo A.R., Silva M.B., Lopes L.C., Piai J.F., Rubira A.F., Muniza E.C. (2012). Hydrogel based on an alginate–Ca^2+^/chondroitin sulfate matrix as a potential colon-specific drug delivery system. RSC Adv..

[31] dos Santos Araújo P., Belini G.B., Mambrini G.P., Yamaji F.M., Waldman W.R. (2019). Thermal degradation of calcium and sodium alginate: A greener synthesis towards calcium oxide micro/nanoparticles. Int. J. Biol. Macromol..

[32] Sánchez-Orozco R., Timoteo-Cruz B., García-Sánchez J.J., Gomez-Espinosa R.M., Bernal-Martínez L.A., Torres-Blancas T. (2024). Properties of eco-friendly orange peel-alginate-glycerol bioplastic films as potential food packaging applications. J. Macromol. Sci. Part A.

[33] Bain E.D., Mrozek R.A., Lenhart J.L. (2017). Role of weak particle-matrix interfacial adhesion in deformation and fracture mechanisms of rigid particulate-filled poly(methyl methacrylate). Mech. Mater..

[34] Lee C.H., Khalina A., Lee S.H. (2021). Importance of interfacial adhesion condition on characterization of plant-fiber-reinforced polymer composites: A review. Polymers.

[35] Baimark Y., Pakkethati K., Srihanam P. (2025). Properties and biodegradation of poly(lactic acid)/thermoplastic alginate biocomposites prepared *via* a melt blending technique. Polymers.

[36] Pathak T.S., Kim J.S., Lee S.-J., Baek D.-J., Paeng K.-J. (2008). Preparation of alginic acid and metal alginate from algae and their comparative study. J. Polym. Environ..

[37] Yang G., Zhang L., Peng T., Zhong W. (2000). Effects of Ca^2+^ bridge cross-linking on structure and pervaporation of cellulose/alginate blend membranes. J. Membr. Sci..

[38] Russo R., Abbate M., Malinconico M., Santagata G. (2010). Effect of polyglycerol and the crosslinking on the physical properties of a blend alginate-hydroxyethylcellulose. Carbohyd. Polym..

[39] Su W., Yang Z., Wang H., Fang J., Li C., Lyu G., Li H. (2022). Synergistic effect of sodium alginate and lignin on the properties of biodegradable poly(vinyl alcohol) mulch films. ACS Sustain. Chem. Eng..

[40] Mascia L., Kouparitsas Y., Nocita D., Bao X. (2020). Antiplasticization of polymer materials: Structural aspects and effects on mechanical and diffusion-controlled properties. Polymers.

[41] Barbut S., Harper B.A. (2019). Dried Ca-alginate films: Effects of glycerol, relative humidity, soy fibers, and carrageenan. LWT-Food Sci. Technol..

[42] Liang X., Ito M., Nakajima K. (2021). Reinforcement mechanism of carbon black-filled rubber nanocomposite as revealed by atomic force microscopy nanomechanics. Polymers.

[43] Zactiti E.M., Kieckbusch T.G. (2006). Potassium sorbate permeability in biodegradable alginate films: Effect of the antimicrobial agent concentration and crosslinking degree. J. Food Eng..

[44] Costa M.J., Marques A.M., Pastrana L.M., Teixeira J.A., Sillankorva S.M., Cerqueira M.A. (2018). Physicochemical properties of alginate-based films: Effect of ionic crosslinking and mannuronic and guluronic acid ratio. Food Hydrocoll..

[45] Olivas G.I., Barbosa-Cánovas G.V. (2008). Alginate–calcium films: Water vapor permeability and mechanical properties as affected by plasticizer and relative humidity. LWT-Food Sci. Technol..

[46] Eslami Z., Elkoun S., Robert M., Adjallé K. (2023). A review of the effect of plasticizers on the physical and mechanical properties of alginate-based films. Molecules.

[47] Minsart M., Vlierberghe S.V., Dubruel P., Mignon A. (2022). Commercial wound dressings for the treatment of exuding wounds: An in-depth physicochemical comparative study. Burn. Trauma.

[48] Xie H., Chen X., Shen X., He Y., Chen W., Luo Q., Ge W., Yuan W., Tang X., Hou D. (2018). Preparation of chitosan-collagen-alginate composite dressing and its promoting effects on wound healing. Int. J. Biol. Macromol..

[49] Tang Z., Fan F., Chu Z., Fan C., Qin Y. (2020). Barrier properties and characterizations of poly(lactic acid)/ZnO nanocomposites. Molecules.

