# Supplementary material for: Reinforcement of Thermo-Compressed Sodium Alginate Films with Calcium Alginate Powder
Source: Mar Drugs. 2026 Apr 17;24(4):142. doi: 10.3390/md24040142 (PMC13117709; doi:10.3390/md24040142)
Supplement: Supplementary file 1 [file marinedrugs-24-00142-s001.zip › marinedrugs-4187256-supplementary.pdf]

Supplementary material

Reinforcement of Thermo-compressed Sodium Alginate Films with Calcium Alginate Powder

Prasong Srihanam [1,*], Wilaiwan Simchuer [2], Vanseng Chounlamany [3], Kesiny Phomkeona [3], Phengxay Deevanhxay [3], and Yodthong Baimark [1,*]

[1] Biodegradable Polymers Research Unit, Department of Chemistry and Centre of Excellence for Innovation in Chemistry (PERCH-CIC), Faculty of Science, Mahasarakham University, Maha Sarakham 44150, Thailand; prasong.s@msu.ac.th (P.S.); yodthong.b@msu.ac.th (Y.B.)

[2] Faculty of Science and Technology, Loei Rajabhat University, Mueang District, Loei 4200, Thailand; wilaiwan.sim@lru.ac.th (W.S.)

[3] Department of Chemistry, Faculty of Natural Sciences, National University of Laos, Vientiane Capital, Vientiane 7322, Laos; v.chounlamany@nuol.edu.la (V.C.); k.phomkeona@nuol.edu.la (K.P.); p.deevanhxay@nuol.edu.la (P.D.)

* Correspondence: prasong.s@msu.ac.th (P.S.); yodthong.b@msu.ac.th (Y.B.)

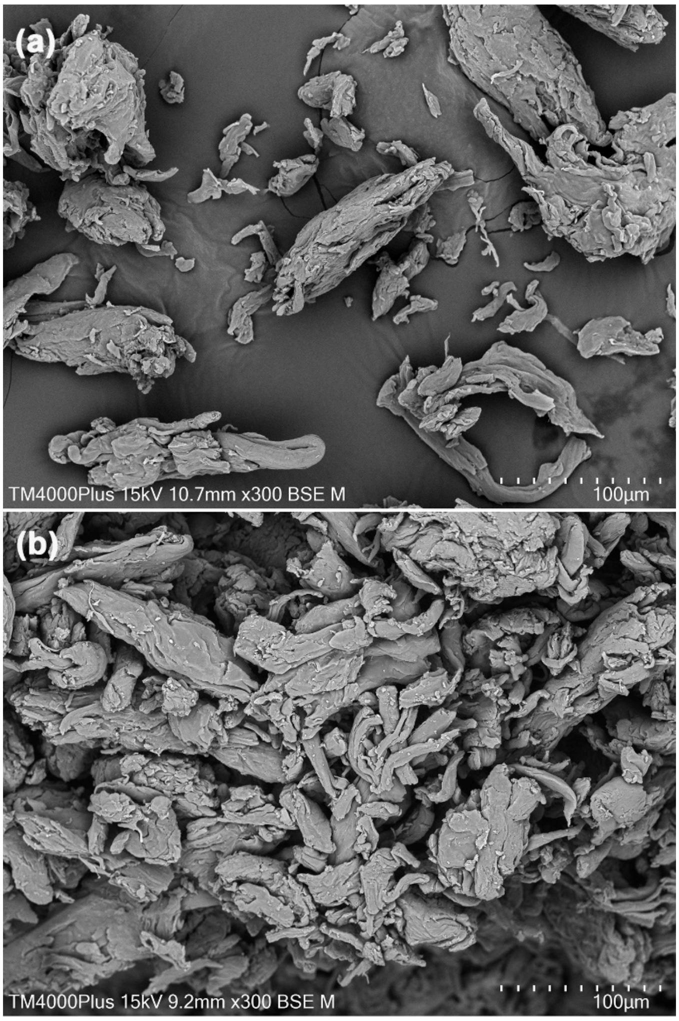

Figure S1. SEM images of (a) CA powder and (b) fractured surface of thermo-compressed CA. All bar scales = 100 μm.

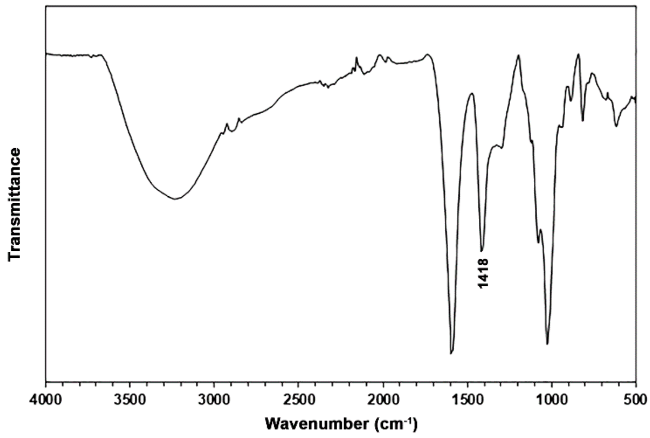

Figure S2. ATR-FTIR spectrum of thermo-compressed CA.

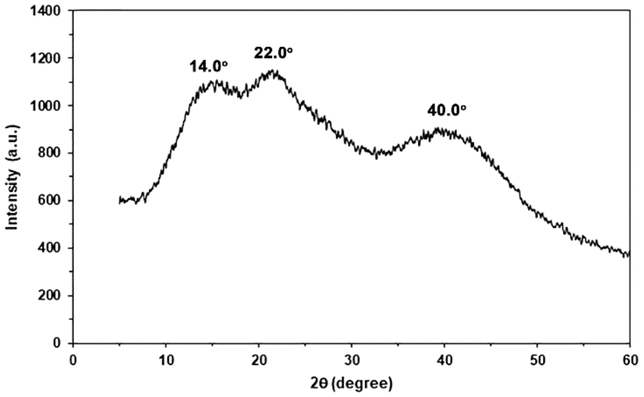

Figure S3. XRD profile of thermo-compressed CA.
